# An Online Universal Diagnosis Procedure Using Two External Flux Sensors Applied to the AC Electrical Rotating Machines

**DOI:** 10.3390/s101110448

**Published:** 2010-11-18

**Authors:** Remus Pusca, Raphael Romary, Andrian Ceban, Jean-François Brudny

**Affiliations:** 1 Univ Lille Nord de France, F-59000 Lille, France; E-Mails: raphael.romary@univ-artois.fr (R.R.); apceban@gmail.com (A.C.); jfrancois.brudny@univ-artois.fr (J.F.B.); 2 LSEE, Faculté des Sciences Appliquées, technoparc futura, 62400 Bethune, France

**Keywords:** AC motors, fault diagnosis, magnetic field sensors, maintenance

## Abstract

This paper presents an original non-invasive procedure for the diagnosis of electromagnetic devices, as well as AC electrical rotating machines using two external flux coil sensors that measure the external magnetic field in the machines’ vicinity. The diagnosis exploits the signal delivered by the two sensors placed in particular positions. Contrary to classical methods using only one sensor, the presented method does not require any knowledge of a presumed machine’s healthy former state. On the other hand, the loading operating is not a disturbing factor but it is used to the fault discrimination. In order to present this procedure, an internal stator inter-turn short-circuit fault is considered as well.

## 1. Introduction

The productivity, reliability and safety of installations using electrical rotating machines are directly influenced by the “healthy” state of these electromechanical converters. Generally, these requirements concerning the operating quality are achieved thanks to an adapted maintenance policy which is often associated with monitoring systems that measure specific parameters like noise, vibrations, temperature or currents [1,2]. The implementation of such systems is expensive and can be justified only in critical cases (power plants). In order to anticipate the failure of a machine, that avoids its replacement or repair during unscheduled periods of maintenance, the machine user needs inexpensive, reliable and easy to implement methods. This aspect justifies the scientific interest to carry out investigations relating to the diagnosis of electrical machines.

Previous works in the field of electrical machine diagnosis are multiple and generally oriented towards the research of specific signatures able to identify or to predict some kind of failure [3–9]. These studies essentially concern the supply current analysis and, more particularly, some harmonic components of the same. Unfortunately, in practical cases, electrical machines are not equipped with convenient systems able to measure and to analyze the current on line. Consequently, noninvasive methods, relating in particular to the exploitation of the data contained in the external magnetic field, can be an alternative to the traditional ones. Another aspect, which characterizes the exploitation of the external magnetic field, concerns the possibility of acquiring information about the failure localization. The studies performed in this field for various kind of machines [10–14], have brought to the fore the specific signatures corresponding to different kind of defects (inter-turn short-circuit in the stator or in the rotor windings, rotor broken bars, eccentricity, *etc*).

Methods which use the external magnetic field of the machine are generally based on the comparison between a reference spectrum corresponding to a healthy state and a given one measured during the machine operating. In some cases, when the fault leads to generate new spectral lines (sideband) then the analysis may be performed without comparison with the healthy signature, but in other cases sensitive indicators may exist even in the healthy machine. Then, their variations, often their increase, give information on the presence of a defect. Moreover, the machine loading operatings, can disturb the diagnosis because it induces other harmonics. These harmonics appear as consequence of the load and they can be confusing with a faulty signature. An additional difficulty is that the presumed healthy state is practically never known before the failure because the machine user has not recorded the corresponding features which characterize the healthy state.

In order to free oneself from these analysis problems, a new noninvasive diagnosis method, which does not require any knowledge of a presumed healthy former state of the machine, is suggested. This method exploits the external magnetic field and more particularly its space variations measured using two flux sensors. Its main interest consists in the fact that the loading operating does not constitute any more, as previously evoked, a perturbing factor but rather it corresponds to an essential state allowing failure discrimination. The procedure presented concerns the detection of a stator inter-turn short-circuit on Salient Synchronous and Induction Machines which will be denoted respectively SSM and IM. The basis of this methodology is an analytical study followed by measurements in both kinds of machines. As a conclusion, the limits of this method are analyzed and commented.

## 2. Sensor Characterization and Principle of the Proposed Diagnosis

### 2.1. Choice of the Sensor

As the method is based on the analysis of the external magnetic field existing in the vicinity of a rotating electrical machine, sensors for magnetic field measurement are used. They can be classified into three categories: based on the Hall Effect, those that exploit the magneto-resistive phenomenon, and those which used specific coils. As the behavior of the external magnetic field harmonic components tied to the slotting effect are considered, the corresponding frequencies are in a medium frequency range going from the hundredth to the thousandth of hertz. Consequently, a coil sensor is more convenient because it induces electromotive force (emf) and this derivative effect amplifies the medium frequencies.

**Figure 1.**
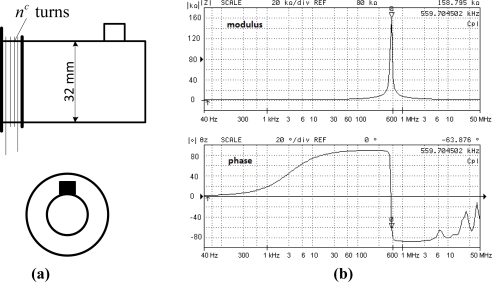
Flux coil sensor: **(a)** design and symbol; **(b)** frequency response.

The used sensor is circular of *S* area (*S* = 8,04*cm*^2^), and the coil is constituted of *n^c^* turns (*n^c^* = 200). Figure 1(a) presents the sensor symbol. Figure 1(b) gives the sensor frequency response, especially the modulus |z| and the phase of the sensor impedance θ_z_ measured with an impedance analyzer. It can be observed that a resonance appears at 559,7 *kHz*. This resonance is tied to the inter-turn capacitive effect combined with the inductive effects. The considered frequency range is lower (some *kHz*) compared to resonance frequency, meaning that this sensor is well suited for the intended application. The drawback of a coil sensor is that it does not give localized information but an average value over the sensor area, so in order to have the most localized information possible, the sensor has to be small compared to the machine size. However, use of a small sensor leads to a decrease of the sensitivity, which can be compensated by increasing *n^c^*, but a high number *n^c^* decreases the resonance frequency. Consequently a compromise has to be made in the choice of sensors.

### 2.2. Principle of the Method

The use of the proposed diagnosis method requires at least two sensors, located symmetrically about the machine axis (180° spatially shifted) and placed close to the motor frame between the end bells, roughly in the middle of the machine. The principle consists in the comparison of the delivered sensor signals according whether the machine runs under no-load or loading conditions. The analysis concerns the magnitude of the specific harmonics of the induced coil emf. One will be interested in the magnitude of the third rank harmonic for SSM and the rotor slotting harmonics for IM. The principle of the method can be described considering a load increase:

▪ if the harmonic amplitudes measured on both sides of a machine vary in the same direction, then the stator winding does not present an inter-turn short circuit fault,
▪ if they vary in opposed directions, then this particular failure can be suspected.

Let us point out that the amplitude of the measured harmonics strongly depends on the fault severity and the location of the sensor in relation to the machine.

## 3. General Considerations

### 3.1. Considerations on the Machine External Field

**Figure 2.**
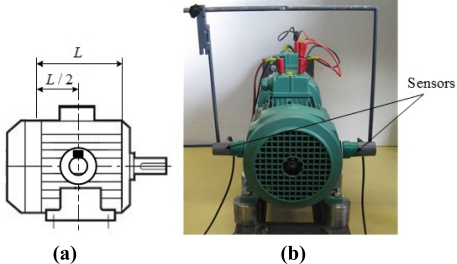
Sensor positions for measuring the transversal external magnetic field: **(a)** position of sensor; **(b)** measurement of external magnetic field.

The stray external magnetic field results from the combination of its axial and transverse components. The axial field is in a plane that contains the machine axis; it is generated by the end overhang effects. The transverse field is located in a plane perpendicular to the machine axis. It is an image of the air-gap flux density *b* which is attenuated by the stator magnetic circuit (sheets package with length *L*) and by the external machine frame. On the other hand, the eddy currents introduce a phase change which differs according to the considered component. It is however possible to measure mainly the transversal field by choosing an adequate position of the sensor such as the effects of the axial field are minimized. This position corresponds roughly to *L*/2 as shown in Figure 2.

In the following, from a theoretical point of view, only the transverse field is considered. In order to identify its harmonic components, the *b* air-gap flux density, which results from the product between the *λ* air-gap permeance and the *ε* resulting magnetomotive force (mmf), has to be determined.

### 3.2. Air-Gap Flux Density Modeling

To determine *b*, the following assumptions are formulated:

- the iron permeability is supposed to be infinite,
- the *p* pole pair, three-phase stator winding, made up of diametrical opening coils, is energized by a balanced three-phase sinusoidal currents system $i_{q}^{s}$ (*q* = 1,2,3) of *I^s^* rms value and *ω* angular frequency.

Let us adopt as space references:

- the *d^s^* axis which is confounded with the stator phase 1 axis,
- the *d^r^* axis which corresponds to one rotor tooth axis.

**Figure 3.**
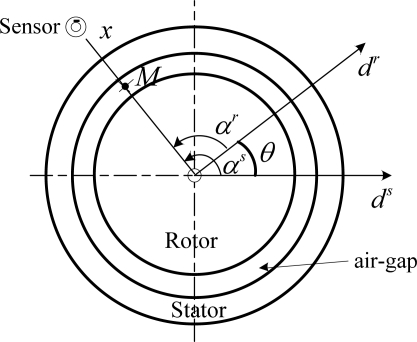
Simplified section of a machine.

Any point *M* in the air-gap can be located by the variables *α^s^* in relation to *d^s^* and *α^r^* in relation to *d^r^* (Figure 3). The axes *d^s^* and *d^r^* are distant of *θ* (the angular gap between the stator and rotor references).

### 3.3. Air-Gap Resulting Magnetomotive Force (mmf)

The *ε^s^* mmf generated by a healthy stator relatively to *d^s^* can be expressed as:

(1) $${ɛ}^{s}=I^{s}\sum_{h^{s}}A_{h^{s}}^{s}\;\text{cos}\left(\omega t-h^{s}p\alpha^{s}\right)$$

where *h^s^* is defined by: *h^s^* = 6*k* + 1 where *k* is an integer number which varies between −∞ to +∞. $A_{h^{s}}^{s}$ is a function that takes into account the winding coefficient tied to the rank *h^s^*.

In the reference frame related to *d^r^*, the *ε^r^* rotor mmf expression, function of *α^r^*, depends on the kind of machine. As *α^r^* = *α^s^* − *θ*, this variable change makes it possible to define *ε*^0^*^r^* which represents the mmf generated by the rotor but defined relative to *d^s^*. Consequently, *ε* is given by:

(2) $$ɛ={ɛ}^{s}+{ɛ}^{0r}$$

### 3.4. Air-Gap Permeance

The *λ* air-gap permeance expressions, different according to the kind of machine, defined relative to *d^s^*, are deduced from the general Equation (3) [15]:

(3) $$\lambda =\sum_{k^{s}=-\infty }^{+\infty }\sum_{k^{r}=-\infty }^{+\infty }\Lambda_{k^{s}k^{r}}\text{cos}\;\;\left[(k^{s}N^{s}+k^{r}N^{r})p\alpha^{s}-pk^{r}N^{r}\theta \right]$$

This relationship concerns an IM taking into account the interaction between the stator and the rotor teeth [16]. *N^s^* and *N^r^* are the stator and rotor per pole pair tooth numbers. Λ*_k^s^k^r^_* is a coefficient which depends on the ranks *k^s^* and *k^r^* and the air-gap geometry. Let *s* denote the slip, *θ* is given by:

(4) $$\theta =\left(1-s\right)\omega t/p+\theta_{0}$$

For the SSM, it suffices to adapt the parameters taking into account that *N^r^* = 2 and *s* = 0.

### 3.5. Air-Gap Flux Density

As *b* = *λε*, the calculus developments lead to define *b* in the reference frame related to *d^s^* as follows:

(5) $$b=\sum_{K,H}b_{K,H}$$

with:

(6) $$b_{K,H}={\hat{b}}_{K,H}\;\text{cos}\left(K\;\omega t-H\alpha^{s}-\varphi_{K,H}\right)$$

An elementary component *b_K,H_* is characterized by two parameters: *K* and *H*. When only one indicator appears in the variable, it corresponds to *K*. A flux density component is thus characterized by its pulsation *Kω* and by its pole pair number *H*.

### 3.6. Transverse External Magnetic Field

The transverse external flux density is deduced from *b* through an attenuation coefficient as previously mentioned. This coefficient, denoted $C_{K,H}^{x}$, is a function of *K, H* and the distance *x* of a point outside of the machine from its axis. Let us point out that for a given *K*, $C_{K,H}^{x}$ decreases when *H* increases [17]. A flux density component of *Kω* angular frequency, denoted $b_{K}^{x}$, is obtained by summation of components having the same frequency rank *K* but different pole pair number *H*. According Equations (5) and (6), at comes:

(7) $$b_{K}^{x}=\sum_{H}b_{K,H}^{x}$$

with:

(8) $$b_{K,H}^{x}={\hat{b}}_{K,H}^{x}\;\text{cos}\left(K\;\omega t-H\alpha^{s}-\varphi_{K,H}^{x}\right)$$

and:

(9) $${\hat{b}}_{K,H}^{x}=C_{K,H}^{x}{\hat{b}}_{K,H}$$

$\varphi_{K,H}^{x}$ results from *φ_K,H_* by taking into account the phase change possibly introduced by the eddy currents.

### 3.7. Measurement

The measurements are performed with a flux sensor presented in section 2.1 placed in *α^s^* = *β*_0_. The *Kω* component of the flux linked by the coil sensor results from:

(10) $$\Psi_{K}^{x}=\int_{S}b_{K}^{x}dS$$

The result of this integration depends of the sensor parameters (*n^c^*, *S*), but also *H* and *x*. Introducing these parameters in the coefficient $K_{H}^{x}$, $\Psi_{K}^{x}$ is expressed by:

(11) $$\Psi_{K}^{x}=\sum_{H}C_{K,H}^{x}K_{H}^{x}{\hat{b}}_{K,H}\;\text{cos}\;\left(K\;\omega \;t-H\beta_{0}-\varphi_{K,H}^{x}\right)$$

Among the components which constitute $\Psi_{K}^{x}$ only few of them, relative to low pole number (low *H*), have a significant contribution [18]. The other components will be absorbed by the ferromagnetic parts of the machine. The *e^x^* delivered by the sensor is given by:

(12) $$e^{x}=\sum_{K}{\hat{e}}_{K}^{x}\;\text{sin}\;\left(K\;\omega t-\varphi_{K,H}^{x}\right)$$

with:

(13) $$\begin{array}{c} {\hat{e}}_{K}^{x}=-K\;\omega \left(\sum_{H}C_{K,H}^{x}K_{H}^{x}{\hat{b}}_{K,H}e^{-j(H\beta_{0}+\varphi_{K,\;H}^{x})}\right)\\ \varphi_{K}^{x}=\mathrm{Arg}\left(\sum_{H}C_{K,H}^{x}K_{H}^{x}{\hat{b}}_{K,H}e^{-j(H\beta_{0}+\varphi_{K,\;H}^{x})}\right) \end{array}\}$$

## 4. Stator Inter-Turn Short-Circuit Modeling

**Figure 4.**
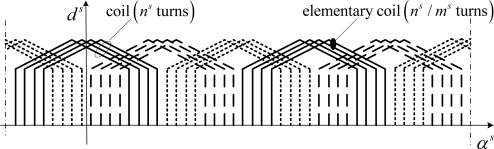
Three phase stator windings (*p* = 2).

Let us consider the *p* = 2, three-phase stator winding given in Figure 4. Each coil is made up of *m^s^* elementary coils of *n^s^* / *m^s^* turns, where *n^s^* is the machine per phase, per pole pair turn number. As the coil of the same phase are series connected it results that the per phase turn number is *pn^s^*.

Let us suppose that *y* turns among *n^s^* / *m^s^* of an elementary coil of the phase *q* are short-circuited. If *y* is small compared to *pn^s^*, it is possible to consider that the $i_{q}^{s}$ currents are unchanged. This assumption makes it possible to characterize the short-circuit by implementing a model which preserves the initial structure of the machine. This model considers that the faulty stator winding is equivalent to the healthy winding, plus *y* short-circuited turn coil which is denoted “additional coil”. In this coil circulates a $i_{qf}^{s}$ fictitious current composed of the short-circuit induced current $i_{sc}^{s}$ and the $i_{q}^{s}$ current in opposite direction:

(14) $$i_{\mathrm{qf}}^{s}=i_{\mathrm{sc}}^{s}-i_{q}^{s}$$

**Figure 5.**
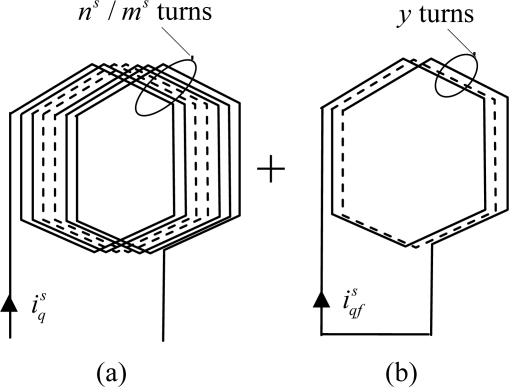
Modeling of an elementary coil with *y* short-circuited turns: **(a)** healthy elementary coil; **(b)** additional coil.

Figure 5 gives a representation of the elementary damaged coil. This approach makes it possible to separate the effects of the fault while preserving the initial structure, what simplifies the fault analysis.

In this way, the resulting air-gap flux density b* can be expressed as:

(15) $$b^{*}=b+b_{\mathrm{qsc}}^{s}$$

**Figure 6.**
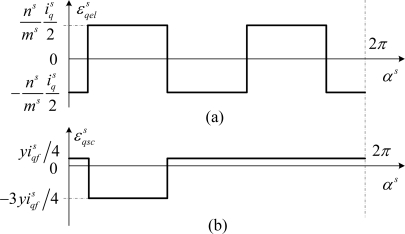
Mmf components generated by elementary damaged coil created by: **(a)** two healthy elementary sections; **(b)** *y* short-circuited turns.

*b* is given by Equation (5) and $b_{\mathrm{qsc}}^{s}$ is generated by the damaged coil. To determine $b_{\mathrm{qsc}}^{s}$, the ${ɛ}_{\mathrm{qsc}}^{s}$ mmf generated by the additional coil must be defined. The ${ɛ}_{\mathrm{qel}}^{s}$ mmf generated by two healthy elementary coils of the same phase, connected in series and shifted of *π*, is presented in Figure 6(a). The Figure 6(b) gives the mmf generated by the faulty turns.

The particularity of this result concerns the ${ɛ}_{\mathrm{qsc}}^{s}$ mmf generated by the additional coil which is a unidirectional wave, although *ɛ^s^* is a rotational one. Another particularity of ${ɛ}_{\mathrm{qsc}}^{s}$ is its pole pair number which is equal to 1, which produces a dissymmetry in the stator mmf wave.

From a mathematical point of view, ${ɛ}_{\mathrm{qsc}}^{s}$ can be written as the sum of rotational mmf waves evolving per pair in opposite directions. In the reference frame related to *d^s^*, ${ɛ}_{\mathrm{qsc}}^{s}$ is expressed by:

(16) $${ɛ}_{\mathrm{qsc}}^{s}=I_{f}^{s}\sum_{h}A_{h}^{s}\;\text{cos}\;\left(\omega \;t-h\alpha^{s}-\varphi_{h}\right)$$

where $I_{f}^{s}$ corresponds to the $i_{\mathrm{qf}}^{s}$ rms value, *φ_h_* is the phase which depends on the position of the elementary coil concerned by the short-circuit but also of the phase where the short-circuit occurs. *h* is a not null relative integer, which takes among others, all the values of *h^s^*.

The flux density $b_{\mathrm{qsc}}^{s}$ generated by the fault is obtained by multiplying ${ɛ}_{\mathrm{qsc}}^{s}$ with *λ*. The step which leads to determine this quantity is identical to that formulated in Section 3.

## 5. Case of a Salient Synchronous Machine

### 5.1. Theoretical Developments

Let us consider a SSM where the rotor winding is energized by a *J* DC current. One assumes that *d^r^* is confounded with a north rotor pole axis. *ε^r^* can be written as follows:

(17) $${ɛ}^{r}=J\sum_{h^{r}}A_{h^{r}}^{r}\;\text{cos}\;\left(h^{r}p\alpha^{r}\right)$$

where *h^r^* takes all odd values varying between 1 and +∞. One can deduce that:

(18) $${ɛ}^{0r}=J\sum_{h^{r}}A_{h^{r}}^{r}\text{cos}\;\left(h^{r}\omega t-h^{r}p\alpha^{s}+h^{r}p\theta_{0}\right)$$

Considering Equations (3) and (4) and taking the particularities that characterize the SSM into account, leads us to express *λ* as:

(19) $$\lambda =\sum_{k^{s}=-\infty }^{+\infty }\sum_{k^{r}=-\infty }^{+\infty }\Lambda_{k^{s}k^{r}}\text{cos}\;\left[2k^{r}\omega t-\left(k^{s}N^{s}+2k^{r}\right)p\alpha^{s}+2k^{r}p\theta_{0}\right]$$

*K* and *H*, which participate in the *b_K,H_* definition given by Equation (6), are defined as:

(20) $$\begin{array}{l} K=h^{r}+2k^{r}\\ H=p\left(h^{\mathrm{sr}}+k^{s}N^{s}+2k^{r}\right) \end{array}\}$$

where *h^sr^* includes all the values taken by *h^s^* and *h^r^*: *h^sr^* = *h^s^* ∪ *h^r^*. One can also deduce that:

(21) $$b_{\mathrm{qsc}}^{s}=\sum_{K_{\mathrm{sc}},H_{\mathrm{sc}}}{\hat{b}}_{sc_{K_{\mathrm{sc}},H_{\mathrm{sc}}}}\;\text{cos}\;\left(K_{\mathrm{sc}}\omega t-H_{\mathrm{sc}}\alpha^{s}-\varphi_{sc_{K_{\mathrm{sc}},H_{\mathrm{sc}}}}\right)$$

with:

(22) $$\begin{array}{l} K_{\mathrm{sc}}=1+2{k^{\prime }}^{r}\\ H_{\mathrm{sc}}=h+p{k^{\prime }}^{s}N^{s}+2p{k^{\prime }}^{r} \end{array}\}\;$$

*k^′s^* and *k^′r^* are equivalent to *k^s^* and *k^r^*. Consequently they vary from −∞ to +∞.

The resulting flux density appears, after attenuation, at the level of the external transverse field. That results in the presence of harmonics at *Kω* and *K_sc_ω* angular frequencies in the signal delivered by the flux sensor. Considering the *K* [Equation (20)] and *K_sc_* [Equation (22)] values, it is possible to make the following remarks:

*- K_sc_* does not bring new frequencies, that means that with the traditional method of diagnosis, the failure presence will be appreciated through the variation of the amplitudes of existing lines in the healthy machine spectrum.
- The sensitive spectral lines are in low frequencies. Indeed, the corresponding harmonics for *k^′r^* = ±1, are *ω* and 3*ω*. Consequently, one of these components is confused with the fundamental [19].
- Other sensitive spectral lines (*kr* = ±2, *kr* = ±3, …) are superimposed on the components at angular frequencies 3*ω*, 5*ω*, 7*ω*, .... that may exist for a healthy machine (supply harmonic, saturation).

These properties make difficult the diagnosis by analysis of the changes in the amplitudes of the measured components. In the following the properties relating to the dissymmetry generated by the fault will be exploited.

### 5.2. Presentation of the Proposed Method

The measurements are carried out in two diametrically opposed positions: position 1 in *α^s^* = *β*_0_ and position 2 in *α^s^* = *β*_0_ + *π*. The principle of the method consists in the analysis of the variations induced by the fault at the level of particular harmonic components measured in the both positions. The corresponding flux density components must have significant amplitude in the external magnetic field. Consequently, taking into account the magnitude of the air-gap flux density and considering the attenuation of these components in the stator core, one will be interested in the components defined by the lowest values of *h^s^*, *h^r^*, *k^s^*, *k^r^*, *k^′s^*, *k^′r^*, leading to the smallest as possible value of *H*.

The respect of these constraints leads to consider the spectral line at *ω* angular frequency (*K* = 1) of magnitude *b̂*_1,1_. However, this component corresponds to the fundamental of the air-gap flux density which generates the main energetic effects: *b̂*_1,1_ ≫ *b̂*_1,_*_H_* with *H* different from 1. It results that *b̂*_1_ will be not very sensitive to the components defined for |*H*|>1. Consequently, the analysis will be focused on the spectral lines related to *K* = 3. If one considers a four pole machine (*p* = 2), the flux density components that contribute to this harmonic are defined as follows:

- for healthy machine: *h^r^* = 1, *k^s^* = 0, *k^r^* = 1, *h^sr^* = 1 in Equation (20), which gives *K* = 3 and *H* = 3 *p* = 6;
- for faulty machine: *h* = −3, *k^′s^* = 0, *k^′r^* = 1 in Equation (22), that leads to *K* = *K_sc_* = 3, *H_sc_* = −1.

Let us consider $b_{3}^{*x}$ the flux density component at 3*ω* angular frequency on the outside of the machine. This component is the sum of a term relating to the healthy machine (of amplitude ${\hat{b}}_{3}^{x}$) and a term relating to the faulty machine (of amplitude ${\hat{b}}_{\mathrm{sc}3}^{x}$). In position 1, the resulting component can be expressed as:

(23) $$b_{3}^{*x(1)}={\hat{b}}_{3}^{x}\;\text{cos}\left(3\omega t-6\beta_{0}+\varphi_{3}^{x}\right)+{\hat{b}}_{\mathrm{sc}3}^{x}\left(\text{cos}3\omega t+\beta_{0}+\varphi_{\mathrm{sc}3}^{x}\right)$$

In position 2, it becomes:

(24) $$b_{3}^{*x(2)}={\hat{b}}_{3}^{x}\text{cos}\left(3\omega t-6\beta_{0}+\varphi_{3}^{x}\right)-{\hat{b}}_{\mathrm{sc}3}^{x}\text{cos}\left(3\omega t+\beta_{0}+\varphi_{\mathrm{sc}3}^{x}\right)$$

The difference between the both positions is tied to the sign which precedes the component due to the fault. For a healthy machine such as ${\hat{b}}_{\;\mathrm{sc}3}^{\;x}=0$, the components defined by (23) and (24) present the same amplitude. In the presence of the fault, the amplitude of the component in one of the positions increases whereas the other one decreases. However, from practical considerations, one can observe differences between measurements for the both positions, even in the healthy case: ${\hat{b}}_{3}^{x(1)}$ in position 1 is different from ${\hat{b}}_{3}^{x(2)}$ in position 2. Two external causes can be responsible for this dissymmetry:

▪ positions 1 and 2 cannot be perfectly symmetrical from each other.
▪ the machine realization techniques can lead to a dissymmetry causing different attenuation coefficients at the level of the two sensor positions.

To solve this problem, the method can be extended thanks to the exploitation of a test in load. The method can be refined as following.

➢ *Under healthy conditions:*

When the machine is loaded, the stator current increases but, at given supply voltage, the air-gap flux density stays practically identical to that obtained at no-load. As the external elements responsible of the attenuation act in the same way in positions 1 and 2 for the no-load and for the load tests, the amplitudes ${\hat{b}}_{3}^{x(1)}$ and ${\hat{B}}_{3}^{x(2)}$ keep similar values or at least evolve in the same way when the machine is loaded.

➢ *Under faulty conditions:*

In loading conditions, ${\hat{b}}_{\mathrm{sc}3}^{x}$ varies taking into account the model chosen to characterize the short-circuit (the loading current contributes to the definition of the fictitious current which circulates in the damaged coil). Consequently, according to (23) and (24), the magnitude of the component at 3*ω* angular frequency in positions 1 and 2 will evolve in opposed directions. The variations of the lines at 3*ω* are thus an indicator of defect. The advantage of the method is that it does not require any knowledge of a healthy state to detect the fault.

### 5.3. Experimental Tests

**Figure 7.**
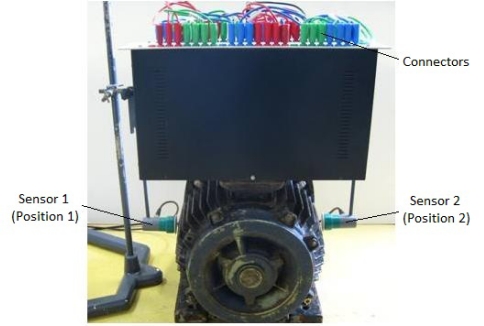
Salient synchronous modified machine and the sensors’ positions.

The tests are carried out on a SSM characterized by: 7.5 *kVA*, 50 *Hz*, *p* = 2, 230 / 400 *V*, *N^s^* = 18. This machine has been rewound so that the terminals of the different stator elementary sections are extracted from the winding and are brought back to a connector block which is fixed above the machine as indicated in Figure 7. So it is possible to produce short-circuits between elementary coils.

The tests carried out when the SSM is connected to the grid, consist in measuring and analyzing the emf delivered by the sensors in order to validate the suggested procedure when an elementary coil is short-circuited (what corresponds to 16, 6% of the whole winding of a phase). In order to avoid damaging the winding, the short-circuit current is limited by an external resistance *R*.

Verification have also be done in order to make sure that the currents in the wires going up from the machine towards the connector block do not disturb the measurements. Positions 1 and 2 correspond respectively to *β*_0_ and *β*_0_ + *π* (see paragraph 5.2) *β*_0_ are chosen “arbitrarily”.

**Figure 8.**
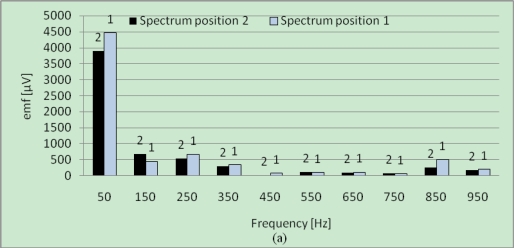

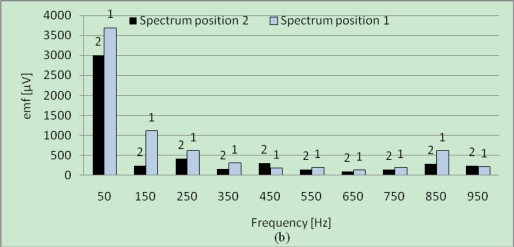
Frequency spectra for SSM at no-load operation for the two positions *β*_0_ and *β*_0_ + *π*: **(a)** healthy machine; **(b)** faulty machine.

Figure 8 presents the sensor emf spectra without [Figure 8(a)] and with [Figure 8(b)] fault at no-load operation in the both positions. As discussed in the theoretical part, it can be observed that a difference appears between the amplitudes of the spectrum lines in positions 1 and 2 for the faulty machine but also in healthy case. It can be also observed that this difference concerning the line at 150 *Hz* is higher in the presence of the defect [Figure 8(b)], with inversion of the predominant line (for the healthy machine, the line in position 2 is higher than this relating to position 1, and inversely in the presence of the defect). However, the inversion of the dominating line as it was expected does not appear for the lines at 50 *Hz*. The loading operation is interesting because it makes it possible to amplify the effect of the fault characterized in theory by the term ${\hat{b}}_{\mathrm{sc}3}^{x}$ in Equations (23) and (24), while keeping on the same level the influence of the natural dissymmetry due to the design of the machine.

**Figure 9.**
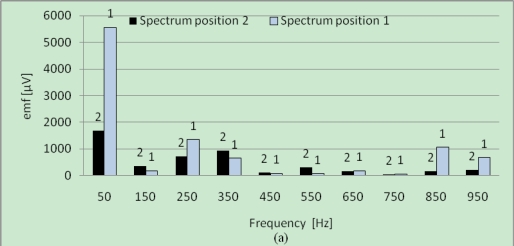

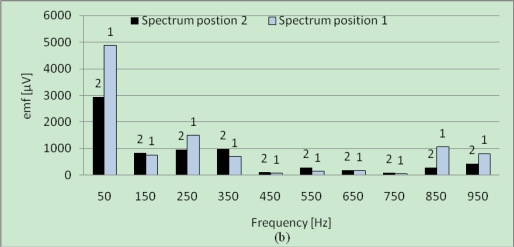
Frequency spectra for SSM at load operation for the two positions *β*_0_ and *β*_0_ + *π*: **(a)** healthy machine; **(b)** faulty machine.

The measured spectra under loading conditions for the healthy machine and the faulty one are given respectively in Figures 9(a,b). It can be observed in Figure 9(a) that the differences between the amplitudes measured in positions 1 and 2 for the lines at 50 *Hz* and 150 *Hz* still exist under loading conditions. The difference is relatively important for the amplitudes of the lines related to the fundamental (50 *Hz*). Figure 9(b), relating to the faulty machine, shows that a small difference characterizes the amplitudes of the lines at 150 *Hz* for the both positions. Consequently, the analysis of the difference between the two positions is not enough to detect the fault because it depends on a high number of parameters: inaccurate position of the sensor, value of the short-circuit current, number of short-circuit turns, state of load, machine’s design, *etc..* In order to minimize these effects, the suggested method consists in combining the tests at no-load and in load operating conditions.

**Figure 10.**
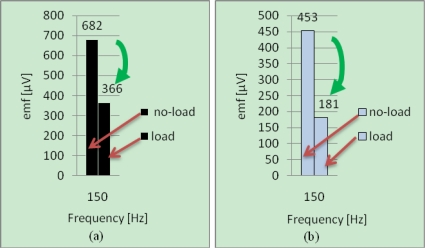
Harmonic components at 150 *Hz* for a healthy SSM at no-load and load operations for the two positions of measurement: **(a)** Position 2; **(b)** Position 1.

Figure 10(a,b) shows the variations of the harmonic at 150 *Hz* relative to the healthy machine when the state (marked with arrow) varies from no-load to loading condition. It can be observed that the magnitudes vary in the same way (decrease) for the two positions.

**Figure 11.**
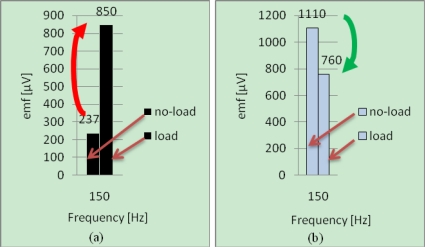
Harmonic components at 150 *Hz* for faulty SSM at no-load and load operations for the two positions of measurement: **(a)** Position 2; **(b)** Position 1.

For the faulty machine the variations of the 150 *Hz* harmonic are given in Figures 11(a,b). It can be seen that the magnitudes in the both positions vary in an opposite way when the machine is loaded. The results are in accordance with the theory, what validates the diagnosis procedure. This procedure has been also test on an interior permanent magnet synchronous machine. The same results were found.

## 6. Case of an Induction Machine

In order to generalize the proposed method, the tests have been also realized on a cage IM characterized by: *p* = 2, 50 *Hz*, 11 *kW*, 380 / 660 *V*, *N^s^* = 24 and *N^r^* = 16. This machine, connected to the grid, has also been rewound like the SSM. Under healthy conditions, the air-gap flux density components are still given by Equations (5) and (6), where the quantities *K* and *H* can be expressed by:

(25) $$\begin{array}{l} K=1+k^{r}N^{r}\left(1-s\right)\\ H=p\left(h^{s}+k^{s}N^{s}+k^{r}N^{r}\right) \end{array}\}$$

The lines sensitive to the fault are characterized by the quantities *K_sc_* and *H_sc_* defined as follows:

(26) $$\begin{array}{l} K_{\mathrm{sc}}=1+{k^{\prime }}^{r}N^{r}\left(1-s\right)\\ H_{\mathrm{sc}}=h+p\left({k^{\prime }}^{s}N^{s}+{k^{\prime }}^{r}N^{r}\right) \end{array}\}$$

According to the values of *N^s^* and *N^r^*, the first sensitive lines to the fault are 750 *Hz* and 850 *Hz* (*kr* = ±1) in the case of null slip. Contrary to the SSM, these lines are located in a frequency range where they can be easily identified. One will be interested to the line at 850 *Hz* rather as this at 750 *Hz* because it is relative to a flux density component having a low pole pair number as well for the healthy case as for the faulty one.

▪ for healthy machine: *k^r^* = 1, *k^s^* = −1, *h^s^* = 7 lead to *K* = 17, *H* = −2 in Equation (25)
▪ for faulty machine: *k^′r^* = 1, *k^′s^* = −1, *h^s^* = 15 lead to *K_sc_* = 17, *H_sc_* = −1 in Equation (26).

Equations similar to (23) and (24) are obtained, but by taking *K* = *K_sc_* = 17 (instead of 3) and *H* = −2 (instead of 6).

**Figure 12.**
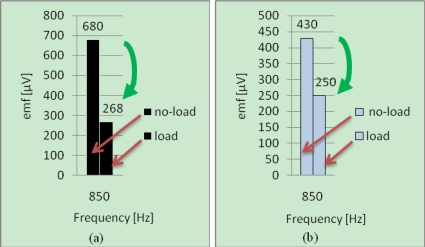
Harmonic components relative to *K* = *K_sc_* = 17 for healthy IM at no-load and load operations for two positions of measurement: **(a)** Position 2; **(b)** Position 1.

**Figure 13.**
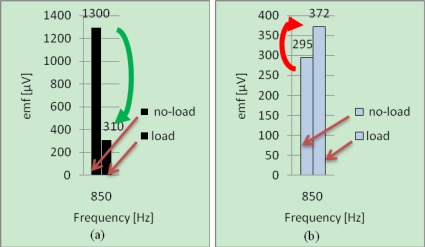
Harmonic components relative to *K* = *K_sc_* = 17 for faulty IM at no-load and load operations for two positions of measurement: **(a)** Position 2; **(b)** Position 1.

The results obtained by considering the harmonic of rank *K* = *K_sc_* = 17 (850 *Hz* at no-load), are presented in Figures 12 and 13.

The phenomena are similar than that obtained on the SSM:

▪ **without fault**–[Figures 12(a,b)]: the lines relative to *K* = *K_sc_* = 17 evolve in the same way with the load,
▪ **with fault**–[Figures 13(a,b)]: these lines evolve in opposite way with the load.

This method is thus applicable to IMs, confirming the universal quality of this diagnosis method.

## 7. Conclusions

The proposed new diagnosis procedure is reliable, inexpensive and simple to implement. This noninvasive method uses two flux coil sensors diametrically located to measure the external magnetic field. This technique eliminates the principal disadvantage of other methods which use only one coil sensor that needs to compare the signature relative this one corresponding to a healthy prerequisite known state. The proposed method presented does not require the knowledge of the healthy signature. It is based on comparison that concerns the state at no-load and load operations.

Actually, this diagnosis procedure can be applied independently if asynchronous or synchronous machine are considered, on condition to choice the suitable spectral component of the observed external magnetic field. This constitutes in our opinion, from an industrial point of view, a considerable added-value.

Practically, all faults can be modeled by a balanced three-phase system to which is added a single-phase system which takes into account the fault. This single-phase system introduces an imbalance on the air-gap magnetic field which is at the origin of the suggested diagnosis procedure. For this reason, this method has been qualified as universal. During the development, implicitly, the need to consider a machine which comprises saliencies on the rotor seems to be a condition in order to apply this method. Studies are at the moment in progress to analyze the possibility of adapting this method for machines with smooth rotor as, for example, synchronous machines with permanent magnets on the rotor surface. Another way of investigations concerns the 2 poles machines.

## Glossary of Symbols

$A_{h^{s}}^{s}$: Function that takes into account the winding coefficient tied to the rank *h^s^*
*α^s^*: Angular gap between the stator reference and a point *M*
*α^r^*: Angular gap between the rotor reference and a point *M*
${\hat{b}}_{3}^{x}$: Amplitude of flux density component for a healthy machine at 3*ω* angular frequency
${\hat{b}}_{\mathrm{sc}3}^{x}$: Amplitude of flux density component for a faulty machine at 3*ω* angular frequency
*b*: Air-gap flux density
*b_K,H_*: Elementary air-gap flux density (is function of *K, H*)
*b**: Resulting air-gap flux density taking into account the fault
$b_{3}^{*x}$: Flux density component at 3*ω* angular frequency
$b_{\mathrm{qsc}}^{s}$: Air-gap flux density generated by the damaged coil
$b_{K}^{x}$: Flux density at distance *x* outside of the machine from its axis
$C_{K,H}^{x}$: Attenuation coefficient in the transversal external magnetic field (is function of *K, H*)
*d^s^*: Axis which is confounded with the stator phase 1 axis
*d^r^*: Axis which corresponds to one tooth axis
*e^x^*: Induced emf delivered by the sensor
*ε^s^*: Stator magnetomotive force (mmf)
*ε^r^*: Rotor magnetomotive force (mmf)
*ε*^0^*^r^*: mmf generated by the rotor but defined relatively to *d^s^*
${ɛ}_{\mathrm{qel}}^{s}$: mmf generated by the healthy elementary coil
${ɛ}_{\mathrm{qsc}}^{s}$: mmf generates by the additional coil
Λ*_k^s^k^r^_*: Coefficient which depends on the ranks *k^s^* and *k^r^* and the air-gap geometry
*λ*: Air-gap permeance
*H*: Poles pair number for *b_K,H_*
*h^s^*: Rank
*I^s^*: RMS value of stator phase current
$i_{q}^{s}$: Instantaneous sinusoidal stator phase current (*q* = 1, 2, 3)
$i_{\mathrm{qf}}^{s}$: Instantaneous fictitious current composed of the *i_sc_* and the $i_{q}^{s}$
*i_sc_*: Short-circuit induced current
*K*: Frequency rank
$K_{H}^{x}$: Sensor coefficient
*k*: Coefficient which varies between −∞ to +∞.
*k^s^*: Coefficient which varies between −∞ to +∞ corresponding to stator
*k^r^*: Coefficient which varies between −∞ to +∞ corresponding to rotor
*L*: Length of sheets package
*M*: Point located in the air-gap
*m^s^*: Number of elementary coils
*n^s^*: Per phase, per pole pair coil turn number
*N^s^*: Stator per pole pair tooth numbers
*N^r^*: Rotor per pole pair tooth numbers
*n^c^*: Sensor turns number
*p*: Pole pair number
$\Psi_{K}^{x}$: Flux component linked by the sensor
*S*: Sensor area
*s*: Slip
*θ*: Angular gap between the stator and rotor references
*ω*: Angular frequency
*x*: Distance of a measure point outside of the machine from its axis
*y*: Number of short-circuited turns
SSM: Salient Synchronous Machine
IM: Induction Machine
